# Diagnosis and Evaluation of Nonalcoholic Fatty Liver Disease

**DOI:** 10.1155/2012/145754

**Published:** 2011-10-27

**Authors:** Mikako Obika, Hirofumi Noguchi

**Affiliations:** ^1^Department of General Internal Medicine, Okayama University Hospital, 2-5-1 Shikata-cho, Okayama 700-8558, Japan; ^2^Department of Gastroenterological Surgery, Okayama University Graduate School of Medicine, Dentistry and Pharmaceutical Sciences, 2-5-1 Shikata-cho, Okayama 700-8558, Japan

## Abstract

Nonalcoholic fatty liver disease (NAFLD) is the most common cause of elevated liver function tests results, after the commonly investigated causes have been excluded, and frequently coexists with type 2 diabetes mellitus (T2DM) because the conditions have common risk factors. As both T2DM and NAFLD are related to adverse outcomes of the other, diagnosis and valuation of fatty liver is an important part of the management of diabetes. Although noninvasive methods, such as biomarkers, panel markers, and imaging, may support a diagnostic evaluation of NAFLD patients, accurate histopathological findings cannot be achieved without a liver biopsy. As it is important to know whether steatohepatitis and liver fibrosis are present for the management of NAFLD, liver biopsy remains the gold standard for NAFLD diagnosis and evaluation. Therefore, new investigations of the pathogenesis of NAFLD are necessary to develop useful biomarkers that could provide a reliable noninvasive alternative to liver biopsy.

## 1. Introduction

Fatty liver, or hepatosteatosis, is characterized histologically by triglyceride accumulation within the cytoplasm of hepatocytes [1] and refers to fat accumulation in the liver exceeding 5%–10% by weight [2]. When hepatosteatosis is present in the absence of excessive alcohol consumption, it is termed non-alcoholic fatty liver disease, or NAFLD [1, 3–5], which is considered to be the hepatic manifestation of the metabolic syndrome [1, 6, 7], a constellation of frequent abnormalities involving insulin resistance, visceral obesity, dyslipidemia, diabetes, hypertension, plus additional factors. Hence, current therapeutic approaches focus on treatment of the underlying risk factors for these metabolic conditions [8]. NAFLD encompasses a spectrum of disorders ranging from simple steatosis to inflammatory steatohepatitis (NASH) and cirrhosis. Of those who develop NASH, about 20% of patients will develop cirrhosis during their lifetime [9]. Therefore, a diagnosis of NASH may result in a more aggressive therapeutic approach toward the metabolic risk factors [10]. In addition, a diagnosis of cirrhosis may suggest the need for an assessment of any associated complications, such as esophageal varices, hepatocellular carcinoma, and so on. The prevalence of NAFLD in the general adult population has been estimated to range from 10% to 24% worldwide; and is as high as 57.5% to 74% in those who are obese [11]. Currently, NAFLD is believed to account for up to 90% of all cases demonstrating elevated liver function test (LFT) results in patients after the commonly investigated causes have been excluded (e.g., viral hepatitis, alcoholism, inherited liver disease, or medications) [11].

**NAFLD and Diabetes**

NAFLD and type 2 diabetes mellitus (T2DM) frequently coexist because they share the risk factors of excess adiposity and insulin resistance. The prevalence of T2DM or impaired fasting glucose ranges from 18–33% in patients with NAFLD, whereas it ranges from 49–62% in T2DM patients who have NAFLD [12–15]. NASH is present in 12.2% of patients with T2DM, as compared to 4.7% in those without T2DM [16]. Moreover, T2DM increases the risk of liver-related death by up to 22-fold as well as overall death by 2.6–3.3-fold in patients with NAFLD [17]. In contrast, the presence of NAFLD among patients with T2DM can also be a risk factor for increased mortality; a community-based study of patients with T2DM revealed that those with NAFLD had 2.2-fold increased risk of mortality compared with those without NAFLD [18]. A recent article also reported that the presence of NAFLD in T2DM patients may also be linked to increased cardiovascular disease (CVD) risk, independently of components of the metabolic syndrome [19, 20], although Ghouri et al. pointed out inconsistencies in the evidence among some articles [21]. Therefore, T2DM is a risk factor for progressive liver disease and mortality in patients with NAFLD, whereas NAFLD may be a marker of cardiovascular risk and mortality in individuals with T2DM. Both T2DM and NAFLD are related to adverse outcomes of the other. In addition, although type 1 diabetes mellitus (T1DM) is due to a relative lack of insulin, an increased prevalence of obesity and insulin resistance in this population means that NAFLD commonly coexists in patients with T1DM [22–25]. An association of NAFLD in T1DM with an increased prevalence of CVD has also been reported [25].

Hence, the diagnosis and evaluation of fatty liver is an important part of the management of diabetes. When diabetes patients are diagnosed with NAFLD, more intensive monitoring and therapeutic intervention are necessary to avoid a poor prognosis. The methods used for the diagnosis and evaluation of NAFLD can also be used to monitor the efficacy of intervention or therapy. In this review, we describe the current trends in the diagnosis and evaluation of NAFLD based on recent articles.

## 2. Diagnosis and Evaluation of NAFLD

The diagnosis of NAFLD needs confirmation of hepatic steatosis based on either imaging studies or liver biopsy, together with the clinical exclusion of individuals who regularly consume >20 g ethanol per day [26]. In the clinical setting, there is still no consensus about whether or not liver biopsy is required to confirm a diagnosis of NAFLD [8].

Presently, the available noninvasive markers for NAFLD include a set of clinical signs and symptoms, laboratory tests, imaging tests, and combinations of clinical and blood test results. Although several of these markers are, in general, useful for the diagnostic evaluation of a patient with suspected NAFLD, they lack the specificity and sensitivity to distinguish NAFL from NASH and to determine the presence and stage of fibrosis [10]. After a diagnosis of NAFLD, the next step is to determine the severity, and this information is necessary to understand the prognosis. Although noninvasive diagnostic methods have advanced recently, a liver biopsy is still required to determine the severity of NAFLD. When staging patients with NAFLD, there are two factors related to the severity: the level of fibrosis and the level of inflammation [27]. We will discuss both invasive and noninvasive means of assessing and staging patients with NAFLD, including information about the characteristics of each method. 

### 2.1. Clinical Features

The majority of patients diagnosed with NAFLD are asymptomatic [8, 28]. When present, clinical symptoms and physical findings are nonspecific and unreliable for diagnosing and assessing disease severity in patients with NAFLD. Patients might have hepatomegaly, general malaise, abdominal discomfort, vague right upper quadrant abdominal pain, nausea, and other nonspecific symptoms referred to the gastrointestinal tract. Clinical examination may reveal ascites, splenomegaly, spider angiomas, palmar erythema, caput medusae, and jaundice in a small percentage of patients who present with NASH-related cirrhosis [8, 28]. The features more consistently found to be associated with disease severity include obesity, older age, diabetes, and hypertension [11].

### 2.2. Common Biomarkers

There is no single biochemical marker that can confirm a diagnosis of NAFLD or distinguish between steatosis, NASH, and cirrhosis [8]. Although mildly elevated serum aminotransferase levels are the primary abnormality seen in patients with NAFLD, liver enzymes may be normal in up to 78% of patients with NAFLD [12, 29]. Additionally, the entire histological spectrum of NAFLD can be observed in patients with normal alanine aminotransferase (ALT) values [30, 31]. Therefore, liver enzyme levels are not sensitive for the diagnosis of NAFLD. The elevations in ALT and aspartate aminotransferase (AST) are typically mild when present and are usually not greater than four times the upper limit of normal [29, 32]. The ratio of AST/ALT is usually less than 1 in patients who have either no or minimal fibrosis, although this ratio may be greater than 1 with the development of cirrhosis [33].

Gamma-glutamyltransferase (GGT) in the serum is frequently elevated in patients with NAFLD, and it has been reported to be associated with increased mortality [34, 35]. However, the diagnosis of NAFLD cannot be made using only GGT. Increased serum GGT levels have also been shown to be associated with advanced fibrosis in NAFLD patients, with a study of 50 NAFLD patients demonstrating an area under the receiver operating characteristic curve (AUROC) of 0.74 for the prediction of advanced fibrosis. Using a cutoff serum GGT value of 96.5 U/L, GGT predicted advanced fibrosis with 83% sensitivity and 69% specificity [36]. Alkaline phosphatase is sometimes slightly elevated, but it is rarely the only liver function test abnormality [37].

When portal hypertension and hepatic synthetic dysfunction are present with cirrhosis, hypoalbuminemia, hyperbilirubinemia, thrombocytopenia, and a prolonged prothrombin time may be seen [28]. Furthermore, an elevated ferritin level has been reported in up to 50% of NASH patients, and elevated transferrin saturation in approximately 10% [33]. However, these findings do not appear to correlate with an elevated iron concentration in the liver, and the role of hepatic iron in the pathogenesis of NASH is unclear [38].

### 2.3. Novel Biomarkers

Several investigators have proposed the measurement of other novel biomarkers to support the diagnosis of NAFLD, but these investigations have been limited by their lack of reproducibility or inability to accurately distinguish simple steatosis from more advanced inflammation and fibrosis [8]. Ideally, novel biomarkers should be helpful for monitoring the progression of NAFLD over time, its response to therapeutic interventions, and for determining the prognosis of the disease. However, there is no such a biomarker available at present [10]. These novel potential biomarkers are shown herein, being classified by the key mechanisms of NASH pathogenesis that they are associated with, including “inflammation,” “fibrosis,” “oxidative stress,” and “hepatocyte apoptosis”. 

#### 2.3.1. Inflammation

A chronic low-grade inflammatory state characteristic of patients with metabolic syndrome has been extensively associated with the development of steatosis as well as liver damage in NAFLD [39, 40]. Inflammatory mediators have also been investigated as potential diagnostic tools. NAFLD is associated with an increase in tumor necrosis factor alpha (TNF-*α*) and decreased adiponectin, and this cytokine imbalance may play an important role in the development of NASH [41–46]. Several groups have reported the circulating levels of these cytokines in the blood from patients with NAFLD to correlate with NASH. Moreover, the TNF-*α* levels were also observed to correlate with the severity of inflammation and fibrosis [41, 47]. However, there are still limited data available on the accuracy and clinical usefulness of these markers for the noninvasive diagnosis of NASH [10]. Shimada et al. reported that the serum adiponectin was significantly lower in patients with early-stage NASH than in those with simple steatosis. Adiponectin had an AUROC of 0.765, sensitivity of 68%, and specificity of 79% for distinguishing early-stage NASH, using a cutoff value of ≤4.0 *μ*g/mL. In this study, the combination of the serum adiponectin level with the Homeostasis Model Assessment-Insulin Resistance (HOMA-IR) level (cutoff value ≥3.0) and type IV collagen 7S (cutoff value ≥5.0 ng/mL) demonstrated a sensitivity of 94% and a specificity of 74% for diagnosing NASH [48]. In another report, using adiponectin and HOMA-IR levels, the AUROC for distinguishing between steatohepatitis and steatosis was 0.79 [43]. However, the relationship between the adiponectin levels and the severity of hepatic fibrosis still remains to be established [49, 50].

The inflammatory marker, C-reactive protein (CRP), lacks specificity for hepatic inflammation and has demonstrated mixed results for NASH. There was a significant increase in high-sensitivity CRP levels in NASH patients compared with controls in some studies [51, 52], and no significant difference in another [53]. Interleukin-6 (IL-6) has also been shown to be elevated in NASH [54]. IL-6 had an AUROC of 0.817 for distinguishing NASH from simple steatosis [55]. The IL-6 levels were also independently related to fibrosis [56]. 

Several authors have reported a relationship to exist between leptin, a hormone secreted from adipose tissue, and liver histology. However, the leptin levels have not been shown to correlate with the degree of steatosis or fibrosis, although some studies have previously reported patients with steatosis and NASH to demonstrate elevated levels of leptin [57–59]. In a recent study, using the combination of HOMA-IR with the adiponectin/leptin ratio, the AUROC was 0.82 for distinguishing between NASH and simple steatosis [56].

Other inflammatory markers, such as CC-chemokine ligand 2 (CCL-2) and hyaluronic acid (HA), have also been shown to be elevated in patients with NASH [60–62]. HA will be described in the next section.

#### 2.3.2. Fibrosis

During the evaluation of NAFLD, it is important to consider the level of fibrosis. Potential fibrosis biomarkers include type IV collagen 7S domain and HA. Sakugawa et al. reported that these two biomarkers were able to exclude advanced fibrosis, with AUROCs of 0.82 and 0.80, and negative predictive values (NPV) of 84% and 78%, respectively, in a cohort of 112 NAFLD cases. These biomarkers also demonstrated positive predictive values (PPV) of 86% and 92% and AUROCs of 0.83 and 0.80 for discriminating between NASH and simple hepatosteatosis [63]. In another report, the AUROC for type IV collagen 7S domain and HA were 0.767 and 0.754, respectively, for the detection of advanced fibrosis in NASH cases, although a multiple regression analysis revealed that only the type IV collagen 7S domain was independently associated with advanced fibrosis in this study [64]. In patients with NAFLD, evaluating the HA levels was found to be useful for predicting severe fibrosis, with an AUC of 0.9, with a cutoff value of serum HA of 46.1 ug/l, yielding a sensitivity of 85% and a specificity of 80% [61]. Furthermore, the platelet count alone was demonstrated to be an independent predictor of cirrhosis, with an AUROC of 0.98 in NAFLD. In this study, HA had an AUROC of 0.97 for detecting severe fibrosis, with a lower AUROC of 0.87 shown for type IV collagen. Serum laminin, which is an extracellular matrix component, was also shown to have an accuracy of 87%, sensitivity 82%, specificity 89%, PPV 82%, and NPV 89% for prediction of fibrosis in NAFLD [65].

#### 2.3.3. Oxidative Stress

Oxidative stress is one of the key mechanisms responsible for liver damage and disease progression in NAFLD [66, 67]. To date, many markers of oxidative stress, including lipid peroxidation products, vitamin E levels, and copper-to-zinc superoxide dismutase and glutathione peroxidase (GSH-Px) activity, have been investigated to determine whether they can be used as surrogate markers of NASH. However, mixed results were demonstrated [68–70], and there is no clear answer at present.

Thioredoxin (TRX) is induced by many oxidative stresses. A significant elevation of the serum TRX levels was demonstrated in patients with NASH in comparison to those with simple steatosis and healthy controls [71, 72].

#### 2.3.4. Hepatocyte Apoptosis

Apoptosis plays an important role in the liver injury observed in NAFLD [73–77]. Recently, a specific byproduct of apoptosis in hepatocytes, caspase-generated cytokeratin-18 (CK-18) fragments, has been shown to be significantly elevated in patients with NASH compared with subjects with fatty liver or healthy controls, with an AUC of 0.93 for predicting NASH [78]. Additionally, Feldstein et al. demonstrated that the plasma CK-18 levels measured using ELISA were significantly higher in patients with biopsy-proven NASH than in those with a borderline diagnosis and normal controls, with an AUROC of 0.83 for NASH diagnosis, in a US multicenter validation study. CK-18 was an independent predictor of both NASH and the severity of disease [79]. Other reported results [78, 80–85] also suggest that CK-18 can be a potentially useful biomarker for the diagnosis and differentiation of NASH from simple hepatosteatosis. CK-18 levels were observed to decrease after bariatric surgery in NASH patients [86]. Therefore, CK-18 fragments might be useful for assessing the response to therapy for NASH. 

The plasma homocysteine levels [87], serum prolidase enzyme activity (SPEA) catalysis [88], plasma pentraxin 3 levels [89], and tissue polypeptide specific antigen [90] are other novel biomarkers for a diagnosis of NASH, however, additional studies are needed to determine their potential for clinical use. 

### 2.4. Panels of Markers

Several studies have been performed to develop noninvasive diagnostic panels and scoring systems that might support the identification of liver steatosis and the diagnosis NASH and to determinate severity of fibrosis, in order to replace the invasive standard liver biopsy. Such scoring systems may potentially represent a more accurate evaluation of global liver fibrosis severity, because the distribution of fibrosis throughout the liver can be uneven in NAFLD [27].

#### 2.4.1. Panel Markers for the Identification of Liver Steatosis

The NAFLD liver fat score includes, as variables, the presence of metabolic syndrome and T2DM, fasting serum insulin, serum AST, and the AST/ALT ratio. This score has an AUROC of 0.86-0.87 to predict liver steatosis, and addition of the genetic information to the score slightly improved the AUROC. Using the same variables, a liver fat equation was developed, from which the liver fat percentage could be estimated [91]. 

 Bedogni et al. developed the fatty liver index (FLI), which uses the body mass index (BMI), waist circumference, triglyceride level, and GGT in the general population with low prevalence of T2DM. This index varies from 0 to 100, and the AUROC was 0.84 to detect liver steatosis [92]. They also reported that the lipid accumulation product (LAP), based on the measurement of waist circumference and the triglyceride level, proved to be a simple and reasonably accurate predictor of ultrasonographic liver steatosis, with an AUROC of 0.8 [93].

 Moreover, the visceral adiposity index (VAI), which uses the BMI, waist circumference, and levels of triglycerides and high-density lipoprotein (HDL) cholesterol, is thought to be capable of indicating both the fat distribution and function. This index was reported to be associated with liver steatosis in patients with chronic hepatitis C, making it a candidate predictor of NAFLD [94].

#### 2.4.2. Panel Markers for NASH Diagnosis

The HAIR (Hypertension, ALT, and Insulin Resistance) score was designed to predict a NASH diagnosis, and includes a combination of the presence of hypertension, elevated ALT, and insulin resistance. The presence of at least 2 parameters predicted NASH with both a high sensitivity and specificity [95]. 

Palekar et al. generated a clinical model to distinguish NASH from simple steatosis by combining 6 different variables including age, gender, AST, BMI, the AST/ALT ratio, and serum HA [96]. The AUROC for this model was 0.76. The presence of 3 or more of these factors had a sensitivity and specificity for a NASH diagnosis of 74% and 66%, respectively. 

Moreover, a simplified model has also been proposed using a logistic regression analysis with only AST and a diagnosis of diabetes, which was able to distinguish NASH from fatty liver with or without nonspecific inflammation in bariatric surgery patients with similar accuracy as the panels described in previous studies [97]. These panels should be thus investigated to validate them in different populations. 

The Nash Test combines 13 biochemical and clinical variables to predict the presence of NASH, achieving specificity, sensitivity, PPV, and NPV of 94%, 33%, 66%, and 81%, respectively [98]. Pelekar et al. combined 8-epi-PGF2_*α*_, TGF-*β*, HA, and adiponectin in a model that predicted NASH with favorable sensitivity of 73.7%, specificity of 65.7%, PPV of 68.2%, and a NPV of 68.2% [96]. 

#### 2.4.3. Panel Markers for Fibrosis in NAFLD

The FibroTest is a validated set of markers for the quantitative assessment of fibrosis, and it includes *α*2-macroglobulin, apolipoprotein A-I, haptoglobin, total bilirubin, GGT, and ALT [99]. The mean standardized AUROC was 0.84 for advanced fibrosis in NAFLD patients after correcting for age and gender in one meta-analysis.

The NAFLD fibrosis score (NFS) is generated using a panel including six variables of age, hyperglycaemia, BMI, platelet count, albumin, and *AST*/*ALT* ratio (AAR), which was created using a large cohort of biopsy-proven NAFLD patients [100]. McPherson et al. reported the NFS to demonstrate an AUROC of 0.81 with NPV 92% and PPV 72% for the detection of advanced fibrosis [101]. Calès et al. demonstrated an AUROC of 0.884 for significant fibrosis, 0.932 for severe fibrosis, and 0.902 for cirrhosis [102]. In a recent meta-analysis, NFS showed AUROC, sensitivity, and specificity of 0.85, 0.90, and 0.97 for the identification of NASH with advanced fibrosis [103]. 

The original ELF (European Liver Fibrosis) test is a panel of automated immunoassays to detect three markers: HA, tissue inhibitor of metalloproteinase 1 (TIMP1), and aminoterminal peptide of procollagen III (P3NP), used in combination with age [104]. The simplified ELF panel excluded age, but did not change the diagnostic performance of the panel. The addition of five markers, including the BMI, presence of diabetes/impaired fasting glucose, AAR, platelet count, and albumin concentration, to the ELF test improved its diagnostic accuracy, with AUROCs of 0.98, 0.93, and 0.84 for the diagnosis of severe, moderate and no fibrosis, respectively [105]. 

The BARD score is a simple scoring system that can be used as a predictive tool in assessing fibrosis in patients with NAFLD. It combines three variables; the BMI, AAR, and the presence of diabetes [106]. A study of the BARD score in 138 patients with biopsy-proven NAFLD demonstrated an AUROC of 0.67, with sensitivity, specificity, PPV, and NPV of 51%, 77%, 45%, and 81%, respectively [107].

The AST-to-platelet ratio index (APRI) was initially used as a marker of fibrosis in patients with hepatitis C [108]. Using this score, Calès et al. reported an AUROC of 0.866 for significant fibrosis, 0.861 for severe fibrosis, and 0.842 for cirrhosis in a study of NAFLD subjects [102], although significantly lower values were obtained in other studies [101, 109, 110].

The AAR [111] is easily calculated using two laboratory liver function tests. Not only is the AAR used as an individual marker, but it is also a component of several other fibrosis scoring systems, including the NFS and BARD score. Using a cut-off of 0.8, AAR alone showed an AUROC of 0.83, with a sensitivity of 74%, specificity of 78%, and a NPV of 93% for the diagnosis of advanced fibrosis in NAFLD [101]. In another study, a combination of serum AST, ALT, and the AAR had an AUROC of 0.59 for predicting steatosis, but was able to predict cirrhosis with an AUROC of 0.81. However, the addition of demographic data, comorbidities, and several other routinely measured laboratory tests increased the AUROCs to 0.79 for NASH and 0.96 for cirrhosis [112].

The components of the FIB-4 test are age with three biochemical values; the platelet count, ALT, and AST, to detect fibrosis [113]. In NAFLD patients, the FIB-4 demonstrated an AUROC of 0.86, sensitivity of 85%, specificity of 65%, and an NPV of 95% for the diagnosis of advanced fibrosis [101]. Interestingly, in a comparison of several markers of fibrosis in NAFLD subjects, FIB-4 had the highest AUROC (0.802) for the diagnosis of advanced fibrosis, followed by the AUROCs for the NAFLD fibrosis score, AAR, APRI, and AST: platelet ratio and BARD score of 0.768, 0.742, 0.73, 0.72 and 0.70, respectively [114]. 

The FibroMeter combines seven variables: age, weight, fasting glucose, AST, ALT, ferritin, and the platelet count [115]. In a study of 235 NAFLD patients, it had AUROCs of 0.943 for significant fibrosis, 0.937 for severe fibrosis, and 0.904 for cirrhosis, respectively. The sensitivity, specificity, PPV, and NPV of the FibroMeter for diagnosing significant fibrosis were 79%, 96%, 88%, and 92% [102]. 

### 2.5. Imaging

Although many imaging tools have been assessed in NAFLD subjects, their main focus has been the quantification of liver fat. The results of these imaging tests cannot be used to differentiate between the histological subtypes of simple steatosis or NASH, nor can they be used to stage the degree of fibrosis [116, 117]. In this section, we explain each imaging modality, while referring to the detection of hepatosteatosis, steatohepatitis, and fibrosis.

#### 2.5.1. Ultrasonography (US)

US is currently the most common method for screening asymptomatic patients with elevated liver enzymes and suspected NAFLD. US findings of fatty liver include hepatomegaly, diffuse increases in the echogenicity of the liver parenchyma, and vascular blunting. Nonsteatotic hepatic parenchyma exhibits an echotexture similar to that of renal parenchyma, but becomes ‘‘brighter” when infiltrated with fat [118]. This hepatorenal contrast can be used for detecting hepatosteatosis [118, 119]. A recent study by Palmentieri et al. of 235 patients undergoing US with liver biopsy showed a sensitivity, specificity, PPV, and NPV of 91%, 93%, 89%, and 94%, respectively, for predicting at least 30% steatosis. However, bright liver contrast was not associated with fibrosis in this study [120]. 

US is easily performed and has a low cost, but it also has some limitations. It is operator dependent and subject to significant intra- and interobserver variability [121]. It is impossible for US to provide quantitative information about the degree of fat accumulation. The sensitivity of US to detect steatosis decreases with a degree of fat infiltration less than 30% [122]. In obese patients, sensitivity lower than 40% has been reported to detect hepatosteatosis [123]. Finally, US has failed to prove efficacious for the detection of inflammation and fibrosis, therefore, it cannot be utilized to diagnose NASH and hepatic fibrosis [10]. In a recent study, however, Iijima et al. used an ultrasound contrast agent (Levovist; Sherling, Berlin) to distinguish between simple steatosis and NASH. Levovist contains galactose and palmitic acid and is taken up by hepatocytes [124]. These moieties participate in the sugar and fat metabolism [125]. The uptake of Levovist is observed to significantly decrease in NASH patients, thus correlating with fibrosis rather than steatosis [124]. Larger studies are needed to evaluate contrast US for use in the diagnosis of NASH and advanced fibrosis. 

#### 2.5.2. Computed Tomography (CT)

CT allows for a more quantitative assessment with measurement of liver attenuation in Hounsfield units (HUs) compared to US, but the information about liver attenuation is not uniform when reported by radiologists. It appears that noncontrast CT scanning is more useful for detecting steatosis than contrast-enhanced scans [126]. Unenhanced CT is more commonly used than enhanced CT [127], and several techniques for determining the appropriate CT values include measurement of hepatic attenuation only [128, 129] and normalization of hepatic attenuation by splenic attenuation, reporting the difference in attenuation between the liver and spleen [127, 129] and the ratio of these values [129, 130]. The attenuation of the spleen is approximately 8–10 HUs less than the liver in normal subjects [127]. With unenhanced CT, an attenuation of the liver is less than 40 HUs [131], or a liver-to-spleen attenuation difference greater than −10 HUs is highly predictive of hepatosteatosis [127]. In addition, a liver-to-spleen ratio of less than 1 is sometimes used to diagnose fatty liver [127, 130]. CT has been demonstrated to be useful for diagnosing >30% steatosis by the use of liver: spleen attenuation ratios; the method has a sensitivity of 73%–100% and a specificity of 95%–100% [29, 132]. The accuracy of unenhanced CT is greatly reduced when there is a lesser degree of steatosis [129]. Other pathologies, such as hepatic siderosis, may also alter attenuation values, thus leading to a misdiagnosis [133, 134]. In longitudinal studies with young subjects, the radiation exposure associated with CT limits its use [133].

#### 2.5.3. Magnetic Resonance Imaging (MRI) and Proton Magnetic Resonance Spectroscopy (MRS)

A good correlation has been reported between MRI, US, and histology in patients with NAFLD [135]. Among these modalities, MRI has been shown to most accurately detect lower levels of steatosis than those detected by US and CT. Fatty changes are assessed by differential chemical shifts between fat and water detected by MRI. MRI was able to detect steatosis of level down to 3% [135]. A variant of MRI, MRS, has also been shown to reliably measure steatosis [136]. Szczepaniak et al. used proton MRS to measure hepatic triglyceride levels (HTGC) in 375 subjects [2]. In this study, 34.3% of the 2,287 participants studied had HTGC >5%, a level deemed diagnostic of hepatic steatosis. MRI and MRS have a higher diagnostic accuracy than US or CT, and MRS can be used to quantify hepatic steatosis. However, none of these imaging techniques have sufficient sensitivity and specificity for staging the disease, and therefore cannot distinguish between fatty liver and NASH with or without fibrosis [116]. Moreover, these tools are more expensive and less accessible than other imaging modalities.

#### 2.5.4. Transient Elastography

Transient elastography (Fibroscan, Echosens, Paris, France) is a non-invasive method of assessing liver fibrosis which can be performed at the bedside or in an outpatient clinic. It uses ultrasound-based technology to measure liver stiffness. Although Fibroscan is less well validated in NAFLD, in a study of 97 NAFLD patients, AUROCs for the diagnosis of significant fibrosis, severe fibrosis, and cirrhosis were reported to be 0.88, 0.91, and 0.99, respectively [137]. Another study including 246 NAFLD patients showed AUROCs for the diagnosis of moderate fibrosis, bridging fibrosis, and cirrhosis of 0.84, 0.93, and 0.95, respectively [138].

The combination of transient elastography with one or more of the serum marker panels might be a potential approach for the non-invasive measurement of fibrosis in NAFLD [10].

### 2.6. Histology

The histological spectrum of NAFLD ranges from simple steatosis through steatohepatitis to fibrosis and cirrhosis [139]. Liver biopsy is the gold standard for diagnosis and has the additional benefit of distinguishing between NASH and simple steatosis, thus allowing for the staging of the degree of fibrosis, which also provides helpful information regarding prognosis and may influence the clinical management of NAFLD [116, 140, 141]. Liver biopsies can also exclude other liver diseases, such as drug-induced hepatotoxicity, Wilson disease and autoimmune hepatitis [11, 142]. Hence, although the diagnosis of NAFLD can usually be confirmed by only a combination of history, serological analyses, and abdominal imaging, liver tissue is needed to determine the severity of NAFLD and to rule out other possible liver diseases [8].

The histological changes in NAFLD are mainly parenchymal and appear in a perivenular location, although portal and periportal lesions may also be present [139]. Simple steatosis is usually macrovesicular, resulting from the accumulation of triglycerides within hepatocytes [143]. NASH requires evidence of lobular inflammation, which usually consists of a mixed mononuclear and neutrophil infiltration. Additionally, hepatocyte ballooning, necrosis, and Mallory's hyaline might be present [144]. Mitochondrial abnormalities may also occur in NASH, but rarely in simple steatosis [145]. As the disease progresses, the typical histological features of steatosis and inflammation frequently disappear and may become completely absent in patients with cirrhosis [146]. Thus, many cases of cryptogenic cirrhosis may be caused by NASH cirrhosis [147–149]. Hepatocellular carcinoma is a complication of NASH-related cirrhosis [117, 150] and also of precirrhotic NAFLD [151, 152]. Other histological findings characteristic of NAFLD in T1DM patients includes diabetic hepatosclerosis and glycogenic hepatopathy [25, 153]. 

 The NAFLD Activity Score (NAS) is used for the histological assessment of NAFLD to distinguish steatosis from NASH in clinical trials [154]. NAS provides a composite score based on the degree of steatosis, lobular inflammation, and hepatocyte ballooning. A score greater than or equal to 5 is likely to represent NASH, a score of 0–2 is unlikely to represent NASH, and a score of 3 or 4 is indeterminate. The NAS does not include fibrosis, and fibrosis is reported separately, on a scale from 0 (without fibrosis) to 4 (cirrhosis) [154]. However, it should be noted that the diagnosis of definite liver steatosis based on evaluations of the patterns on liver biopsy does not always correlate with the threshold values of the semiquantitative NAS, which was developed as a tool to measure changes in NAFLD during therapeutic trials [155].

**Limitations of Liver Biopsy**

There are several limitations associated with liver biopsy. Liver biopsy, being an invasive procedure, has a small risk of complications including pain, bleeding, and, rarely, death. Next, since only a very small portion of the liver is obtained from the needle liver biopsy as a tissue sample (1/50,000 of the total mass of the liver), the biopsy is prone to significant sampling error [156, 157], especially regarding such features as fibrosis, which are often not uniformly distributed. Finally, another important limitation of liver biopsy is the fact that the histological analysis remains subjective, is influenced by the skill and experience of the reading pathologist, and is thus prone to intra- and interobserver variability [154, 158].

## 3. Conclusion

At present, NAFLD is the most common cause of elevated LFT results, after the commonly investigated causes have been excluded. NAFLD and T2DM frequently coexist because of their similar pathogenic abnormalities. As both T2DM and NAFLD are related to adverse outcomes of the other, the diagnosis and evaluation of fatty liver is an important part of the management of diabetes. Noninvasive methods are favorable ways to support a diagnosis of hepatosetaotsis, but accurate histopathological findings and staging of fibrosis cannot be achieved without a liver biopsy. It is important to determine whether NASH and liver fibrosis are present, because close monitoring and followup are necessary for these patients. Therefore, a liver biopsy remains the gold standard for the diagnosis and evaluation of NAFLD. However, new investigations on the pathogenesis of the disease progression in NAFLD might result in the development of useful biomarkers that could provide a reliable noninvasive alternative to liver biopsy.
